# Disease-modifying therapeutic strategies in osteoarthritis: current status and future directions

**DOI:** 10.1038/s12276-021-00710-y

**Published:** 2021-11-30

**Authors:** Yongsik Cho, Sumin Jeong, Hyeonkyeong Kim, Donghyun Kang, Jeeyeon Lee, Seung-Baik Kang, Jin-Hong Kim

**Affiliations:** 1 Department of Biological Sciences, College of Natural Sciences, Seoul National University, Seoul, 08826 South Korea; 2 Center for RNA Research, Institute for Basic Science, Seoul, 08826 South Korea; 3 Department of Business Administration, Business School, Seoul National University, Seoul, 08826 South Korea; 4 Department of Orthopaedic Surgery, Seoul National University College of Medicine, Boramae Hospital, Seoul, 07061 South Korea; 5 Interdisciplinary Program in Bioinformatics, Seoul National University, Seoul, 08826 South Korea

**Keywords:** Osteoarthritis, Translational research

## Abstract

Osteoarthritis (OA) is the most common form of arthritis. It is characterized by progressive destruction of articular cartilage and the development of chronic pain and constitutes a considerable socioeconomic burden. Currently, pharmacological treatments mostly aim to relieve the OA symptoms associated with inflammation and pain. However, with increasing understanding of OA pathology, several potential therapeutic targets have been identified, enabling the development of disease-modifying OA drugs (DMOADs). By targeting inflammatory cytokines, matrix-degrading enzymes, the Wnt pathway, and OA-associated pain, DMOADs successfully modulate the degenerative changes in osteoarthritic cartilage. Moreover, regenerative approaches aim to counterbalance the loss of cartilage matrix by stimulating chondrogenesis in endogenous stem cells and matrix anabolism in chondrocytes. Emerging strategies include the development of senolytic drugs or RNA therapeutics to eliminate the cellular or molecular sources of factors driving OA. This review describes the current developmental status of DMOADs and the corresponding results from preclinical and clinical trials and discusses the potential of emerging therapeutic approaches to treat OA.

## Osteoarthritis: moving from symptom relief towards disease control

Researchers are exploring several promising avenues to develop treatments for osteoarthritis that control or reverse its progress, rather than simply relieving the symptoms of inflammation and pain. These new opportunities are emerging from increased understanding of the molec_reular mechanisms underlying the onset and progression of osteoarthritis. Seung-Baik Kang, Jin-Hong Kim and colleagues at Seoul National University, South Korea, review the current status and future directions of disease-modifying strategies that are already showing promise in clinical trials. They discuss approaches aimed at restricting the damage to joint cartilage by controlling the inflammatory signaling molecules, enzyme actions and molecular pathways that cause cartilage to degenerate. They also consider treatments that could stimulate the active regeneration of damaged cartilage. They conclude that some approaches will soon be ready to move into general clinical use.

## Introduction

The key feature of osteoarthritis (OA) is the gradual loss of articular cartilage. Other OA-related manifestations include osteophyte formation at joint margins and bone remodeling that accompanies bone marrow lesions and subchondral bone sclerosis^1–4^. Synovial inflammation and meniscal damage are common features of OA. All of these OA manifestations collectively lead to the impairment of joint function and the development of chronic pain, and OA is widely considered a whole-joint disease^5^.

OA treatment has been largely limited to steroidal or nonsteroidal anti‐inflammatory drugs that provide symptomatic relief from pain and inflammation^6^. Next-generation OA treatments, often referred to as disease-modifying OA drugs (DMOADs), are under development and aim to modify the underlying OA pathophysiology and alleviate the associated structural damage to prevent long-term disability. Although DMOADs are not yet available in the pharmaceutical market, several clinical trials are ongoing^7^. One group of promising DMOADs delays cartilage degeneration by targeting pro-inflammatory cytokines, the proteolytic activities of catabolic enzymes, and the Wnt pathway. Another group of drugs stimulates the regenerative potential of cartilage to counteract matrix loss in osteoarthritic cartilage. The emerging DMOAD therapies under active investigation aim to eliminate senescent chondrocytes or use RNA-based approaches to modulate OA-inducing mechanisms.

## DMOADs based on the molecular mechanisms underlying OA pathogenesis

Based on recent advances in our understanding of the mechanisms underlying OA pathogenesis, various DMOADs have been developed. In particular, an imbalance between matrix anabolism and catabolism contributes to osteoarthritic cartilage degeneration^4,8^. The DMOADs that are currently in clinical trials aim to restore the homeostasis of matrix metabolism.

### Pro-inflammatory cytokines and matrix-degrading enzymes

Therapeutic strategies targeting pro-inflammatory cytokines, matrix-degrading enzymes, or Wnt signaling have been developed to delay the catabolism of cartilage matrix in OA patients.

### Targeting pro-inflammatory cytokines

**Fig. 1:**
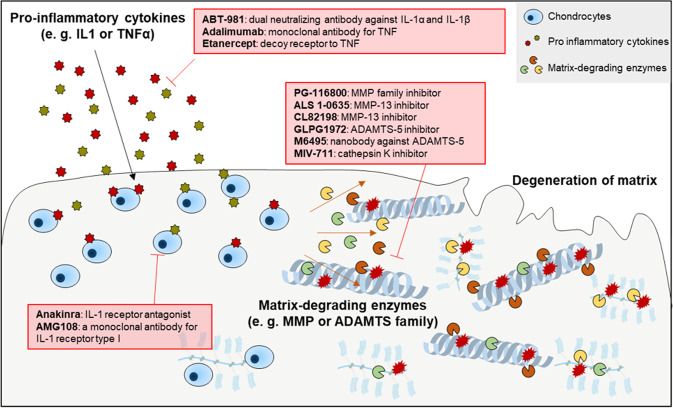
Pharmacological management of OA by blocking pro-inflammatory cytokines and matrix-degrading enzymes. IL-1 and TNF are the major pro-inflammatory cytokines that stimulate the production of matrix-degrading enzymes and inflammatory mediators in joint tissues. MMP and ADAMTS family members degrade the extracellular matrix components of cartilage, promoting osteoarthritic cartilage destruction.

Interleukin (IL)-1 and tumor necrosis factor (TNF) are the most well-characterized pro-inflammatory cytokines and stimulate the production of inflammatory mediators, such as prostaglandin E, nitric oxide synthase, chemokines, and other cytokines, in the joint microenvironment^9–13^. Furthermore, IL-1 and TNF directly promote the expression of matrix metalloproteinases (MMPs) and other matrix-degrading enzymes involved in cartilage degeneration^9,10^. Therefore, there have been rigorous attempts to treat OA by inhibiting the IL-1 and TNF pathways (Fig. 1). However, the results of clinical trials of therapeutic candidates that block these pro-inflammatory cytokines have been rather unsatisfactory despite the fact that these candidates effectively suppress the inflammatory phenotypes in chondrocytes in vitro^14^. Intra-articular injection of anakinra, an IL-1 receptor antagonist that obstructs the receptor binding of both IL-1α and IL-1β, into 160 individuals with knee OA did not reduce OA-associated pain or cartilage turnover during weeks 4–12 of administration in a random controlled trial (NCT00110916, phase II clinical trial)^15^. Likewise, a randomized double-blind controlled trial (NCT00110942, phase II clinical trial) of AMG108, which is a monoclonal antibody against IL-1 receptor type I that blocks the receptor binding of both IL-1α and IL-1β, did not provide sufficient clinical benefits^16^. ABT-981 (a dual neutralizing antibody against IL-1α and IL-1β) was tested in patients with hand^17^ or knee^18^ OA. Neither two phase II clinical trial (NCT02384538 and NCT02087904) showed substantially improved outcomes, indicating that ABT-981 is ineffective in treating OA. In a clinical trial involving 43 hand OA patients with random allocation to groups administered adalimumab (TNF antibody) or placebo for 12 weeks, no significant difference in hand pain was noted between the two groups^19^. Similarly, in a trial of 90 patients with hand OA, etanercept (a decoy receptor that binds to TNF) did not differ from placebo in alleviating pain after 24 weeks of administration^20^.

### Targeting matrix-degrading enzymes

MMPs are a family of zinc-dependent proteolytic enzymes that degrade the components of the extracellular matrix^21^. Various MMPs are upregulated in the degenerating cartilage of OA patients^22,23^. Although some of the developed MMP inhibitors have shown notable effects on preclinical OA models^24–28^, only a few have entered clinical trials for patients with mild-to-moderate knee OA (Fig. 1).

The clinical efficacy of PG-116800, a small-molecule inhibitor with a high affinity for MMP-2, -3, -8, -9, -13, and -14, was tested in 401 patients with knee OA with random allocation to treatment groups that also included a placebo group^29^. No statistically significant difference in knee-joint space width or the Western Ontario and McMaster Universities Osteoarthritis Index WOMAC score was observed between the test and placebo groups. Furthermore, some side effects, such as restricted joint motion and arthralgia, were observed in the test group^29^. Although the cause of these adverse effects remains unclear, MMP inhibitors may broadly affect the matrix turnover in musculoskeletal tissues other than cartilage^30^. Accordingly, these studies provide evidence that broad-spectrum MMP inhibitors are unlikely to be suitable for OA treatment due to their side effects.

MMP-13 has attracted the most attention as a promising therapeutic target because it has the highest substrate specificity against type II collagen, the most abundantly present collagen in cartilage. Wang et al. examined the effect of CL82198, a specific MMP-13 inhibitor, on inhibiting MMP-13 activity in a preclinical model of OA^24^. In mice with surgically induced OA, different doses of CL82198 or control saline was intraperitoneally injected daily beginning 1 day after the surgery. OA progression was significantly alleviated after CL82198 administration. No follow-up clinical studies on this compound have been performed yet. Recently, Baragi et al. developed the MMP-13 inhibitor ALS 1-0635 and evaluated its efficacy in an OA rat model^31^. The researchers orally administered ALS 1-0635 to rats twice a day for 3 weeks and found that ALS 1-0635 protected the cartilage from osteoarthritic destruction. Of note, frequent administration of the relatively high dose of 60 mg/kg ALS 1-0635 was effective, suggesting potential shortcomings associated with the low substrate specificity of ALS 1-0635.

ADAMTS-4 and -5 are principal enzymes responsible for cleaving aggrecan, the major proteoglycan in articular cartilage. Knockout of *Adamts5* but not *Adamts4* in mice alleviated OA-induced proteoglycan loss in cartilage, suggesting that ADAMTS-5 is the primary enzyme responsible for aggrecan cleavage^32,33^. GLPG1972 is a highly selective, orally bioavailable small molecule that inhibits ADAMTS-5^34^ (Fig. 1). Two phase I studies (NCT02851485 and NCT03311009) showed that GLPG1972 was safe and well tolerated without any evident adverse events^35,36^. The drug caused a decrease up to 53% decrease in the serum levels of the aggrecan neo-epitope generated by ADAMTS-5 catalytic activities. Unfortunately, a recent phase II study (NCT03595618) with 938 patients did not meet the primary endpoint, pending detailed results to be reported^37^. Another novel ADAMTS-5 inhibitor under development is nanobody M6495. Nanobodies are single-domain monoclonal antibodies whose antigen-binding sites are composed of one heavy chain; thus nanobodies are markedly smaller in size than conventional monoclonal antibodies^38^. M6495 is a bifunctional nanobody that can bind to both ADAMTS-5 metalloproteinase/disintegrin domains and human serum albumin (Fig. 1). The binding of M6495 with albumin extends its half-life in vivo^39^. In a phase I clinical trial (NCT03224702), M6495 was subcutaneously injected into healthy male subjects and demonstrated an acceptable safety and tolerability profile^40^. Another phase I study (NCT03583346) was conducted to validate the safety and efficacy profile in OA patients. The results are expected to be announced in the near future.

The cysteine cathepsin family is composed of eleven members^41,42^. Cathepsins B, H, K, L, and S are the best-known members of the cathepsin family and can degrade native collagens and other components of the ECM^43–45^. In particular, increased expression of cathepsin K has been observed in the degenerative cartilage of human OA^46,47^. Multiple selective cathepsin K inhibitors have been shown to be effective in treating OA in animal models, ameliorating cartilage degeneration^48,49^ or joint pain^50,51^. MIV-711, an orally administered small-molecule cathepsin K inhibitor, is in clinical development for OA treatment (Fig. 1). In a phase I trial (NCT03443453) evaluating bioavailability, MIV-711 was found to be safe and well tolerated in healthy subjects^52^. MIV-711 did not meet the primary endpoint for the Numeric Rating Scale (NRS) knee pain score in the phase II clinical trial and its extension substudy (NCT02705625 and NCT03037489). Nevertheless, MIV-711 has shown some beneficial effects in terms of cartilage thickness, as assessed by radiological analysis, and OA-associated pain measured according to WOMAC^53–55^, leaving room to further improve the clinical efficacy of cathepsin K inhibitors.

### The Wnt pathway

The Wnt signaling pathway is transduced through a large family of Wnt glycoproteins (19 genes in mammals)^56^. β-Catenin is one of the important protein in canonical Wnt signaling, which regulates the development and homeostasis of joints^57^. Activation of the Wnt signaling pathway has been noted in the cartilage, bone, and synovial membrane in OA patients^58,59^.

Canonical Wnt signaling starts with Wnt binding to Frizzled receptors, leading to the disruption of the β-catenin destruction complex. Stabilized β-catenin then translocates into the nucleus and interacts with the transcription factors T-cell factor (TCF) and lymphoid enhancer factor (LEF), activating the expression of Wnt target genes^60,61^. Interestingly, β-catenin levels are frequently upregulated in OA joint tissues, causing chondrocyte hypertrophy and synovial inflammation^62–66^. Canonical Wnt signaling plays an essential role in regulating bone remodeling and repair^57^, indicating that this signaling pathway needs to be carefully modulated in the joint when developing Wnt-targeting therapeutic strategies. Indeed, previous strategies targeting members of the Wnt pathway, such as β-catenin or upstream members, have not resulted in FDA-approved drugs^67,68^, suggesting that the selective regulation of Wnt target genes or approaches that spare β-catenin may be necessary.

Notably, lorecivivint (also known as SM04690) was identified through high-throughput screening for compounds targeting the Wnt signaling pathways and demonstrated efficacy in mitigating cartilage degeneration in a rat model of OA^69^. Later, the anti-inflammatory and chondroprotective effects of lorecivivint were found to be unrelated to β-catenin but were mediated by the inhibition of two intranuclear kinases, CLK2 and DYRK1A^70^. In a phase I trial (NCT02095548) involving 61 patients with moderate-to-severe knee OA, intra-articular administration of lorecivivint effectively restricted systemic exposure of lorecivivint and did not induce any severe adverse events, thus validating the safety of this compound^71^. In a phase IIa proof-of-concept study (NCT02536833) involving 455 patients, compared with placebo treatment, lorecivivint treatment did not meet the primary endpoint of improvement set by the WOMAC pain score by week 13^72^. However, at week 52, patients treated with the 0.07 mg dose showed significant improvements compared with those in the placebo group^72^. In a phase IIb study (NCT03122860), among the 695 patients treated with either of four different doses (0.03, 0.07, 0.15, and 0.23 mg), those treated with 0.07 and 0.23 mg showed statistically significant improvements in OA-associated pain according to the NRS and WOMAC pain score^73^. The phase II clinical trial (NCT03706521) was completed in December 2020, but the results had not yet been reported when this review was prepared. Other ongoing or scheduled clinical trials (Phase II: NCT03727022 and Phase III: NCT03928184, NCT04385303, and NCT04520607) are underway to test the efficacy of long-term administration of lorecivivint at the optimized dose of 0.07 mg.

### Cartilage regeneration

DMOADs targeting catabolic factors are effective in delaying further cartilage degeneration but are insufficient in reconstructing degenerated tissue. Regenerative therapy aims to restore the normal architecture and function of a damaged joint. Cartilage regeneration is mediated by the chondrogenic differentiation of stem cells and the synthesis of cartilage matrix by chondrocytes^3,74^. However, the regenerative capacity of cartilage tissue in joints markedly declines with age and traumatic joint injuries.

Kartogenin is a small molecule that stimulates chondrogenic differentiation in mesenchymal stem cells (MSCs) and was developed by Johnson et al. in 2012 for the purpose of cartilage regeneration^74^. Kartogenin binds to filamin A and consequently interrupts the interaction of filamin A with the transcription factor core-binding factor β subunit, thereby upregulating type II collagen and aggrecan expression^74^. While kartogenin showed promise in stimulating cartilage regeneration, several challenges remain in its clinical applications. Recently, through extensive medicinal modifications, KA34^75^ was developed as an analog of kartogenin, and this variant significantly improved the potency and chemical stability of kartogenin. With an improved safety and efficacy profile, KA34 has recently finished a phase I clinical study (NCT03133676) with 60 OA patients, but the results have not yet been reported.

LNA043 is a novel angiopoietin-like protein 3 (ANGPTL3) agonist^76^. Human ANGPTL3 is a 460-amino-acid polypeptide that is mainly involved in regulating lipid metabolism and angiogenesis^77^. The current patent (US20160213748A1) claims a novel role of ANGPTL3 in facilitating the chondrogenic differentiation of MSCs. An ANGPTL3-variant polypeptide has been shown to enhance chondrogenesis, playing a chondroprotective role in a preclinical OA mouse model (US20160213748A1, WO2014138687A1). A phase I clinical trial (NCT03334812) of LNA043 in patients with knee cartilage defects was completed early based on favorable outcomes in terms of safety and tolerability. An additional phase I study (NCT02491281) of knee OA patients further confirmed the safety of LNA043 without eliciting any noticeable immune responses. The researchers also showed that the compound was effectively delivered by penetrating the cartilage layers, enhancing the anabolic activities of cartilage. Patients with cartilage lesions and knee OA are currently being recruited for the phase II trial (NCT03275064) of LNA043.

Tankyrase inhibition has been suggested as a potential strategy to simulate regenerative potentials in osteoarthritic cartilage^3^. Pharmacological inhibition of tankyrase induces chondrogenic differentiation in MSCs and stimulates the expression of cartilage-specific matrisome, collectively ameliorating osteoarthritic cartilage destruction in preclinical models of OA^3^. Recent accomplishments in fostering the regenerative capacity of adult cartilage suggest the clinical potential of regenerative therapy as an OA treatment.

### OA-associated pain

Chronic pain is one of the prominent symptoms of OA, and the clinical management of OA largely aims pain relief. Molecular pathways eliciting chronic pain are regulated in a complex manner via the peripheral and central nervous systems. Although cartilage is an aneural tissue, nociceptors are abundant in other tissues of the joints, such as the joint capsule, synovium, subchondral bone, and ligaments^78^. Specific receptors on the peripheral terminal, such as heat receptors, chemoreceptors, and mechanoreceptors, detect diverse stimuli, including cytokines, chemokines, neuropeptides, and prostaglandins^78,79^. These factors form a biochemical milieu that elicit OA-associated pain in the joint. With the progression of peripheral sensitization, joint movement within the normal range becomes painful. Central sensitization also contributes to an abnormal state of responsiveness or increased gain in the nociceptive system^80^. Collectively, OA patients experience hypersensitivity to noxious stimuli, which is generally characterized by mechanical allodynia or hyperalgesia^81,82^. There have been recent advances in understanding the cellular and molecular basis of mechanical allodynia and hyperalgesia development in OA-affected joints. The critical role of nerve growth factor (NGF) in damaged joint environments has been linked to pain development in OA patients^83,84^.

NGF is a member of neurotrophins in the peripheral and central nervous system^85^. On peripheral nociceptors, the interaction of NGF and its receptor, tropomyosin-related kinase A (TrkA), activates transient receptor potential cation channel subfamily V member 1 (TRPV1) and contributes to pain hypersensitivity associated with tissue damage^86,87^. NGF expression is elevated in various cell types (e.g., synoviocytes, chondrocytes, osteoclasts, and some immune cells) in the synovium, cartilage, and subchondral bone in patients with knee OA^85,88,89^. Therefore, NGF has been suggested to be a rational target whose inhibition may effectively manage OA-associated pain in joints^78^.

Tanezumab is a humanized IgG2 monoclonal NGF antibody that effectively interferes with the binding of NGF to its corresponding receptors^90^. Phase II clinical trials (NCT00394563) showed that a single intravenous injection of tanezumab substantially reduced pain in patients with knee OA^83^. A randomized phase III clinical study (NCT02709486) with a 24-week follow-up period demonstrated the significant efficacy of subcutaneously injected tanezumab in controlling OA-associated pain in the hip or knee^91^. However, safety concerns have been raised recently, along with the report that tanezumab increases the onset of rapidly progressive OA and abnormal peripheral sensation^92^.

Fasinumab, a human monoclonal NGF antibody^93^, has been tested in multiple clinical phase trials involving patients with knee or hip OA. A phase IIb/III double‐blind clinical trial (NCT02447276) was conducted with 421 patients with moderate-to-severe knee or hip OA, and 346 patients completed the study^94^. Patients were randomized to receive 1, 3, 6, or 9 mg fasinumab or placebo which was administered subcutaneously every 4 weeks for 16 weeks with a 36-week follow-up. Fasinumab induced significant reductions in OA-associated pain and improvements in physical function for patients with OA. A phase III clinical trial (NCT02683239) has been conducted to test the long-term safety and efficacy of fasinumab in knee or hip OA patients, but the results have not yet been reported.

Fulranumab, another human monoclonal antibody against NGF^95^, underwent a phase II clinical trial (NCT01094262) involving 196 patients with moderate-to-severe chronic knee OA. Patients were subcutaneously injected with 3 or 9 mg fulranumab or placebo every 4 weeks for 12 weeks. Fulranumab administration improved the NRS knee pain score compared with that of the active comparator oxycodone^96^. In another phase II clinical trial (NCT00973141), patients with knee or hip OA were randomized to receive subcutaneous injections of placebo or various doses of fulranumab. Knee and hip pain, as assessed by the WOMAC score, were effectively alleviated by fulranumab administration (3 mg every 4 weeks or 10 mg every 8 weeks) as early as 4 weeks, and the effect was maintained for up to 53 weeks. However, rapidly progressive OA was observed as an adverse effect^97^. In phase III clinical trials (NCT02336685, NCT02336698, NCT02289716, and NCT02301234), patients with moderate-to-severe OA were randomized to receive subcutaneous injections of placebo or fulranumab (1 or 3 mg every 4 weeks) in the 16-week double-blind phase, followed by a 52-week posttreatment follow-up phase. Fulranumab improved pain management and physical function in patients with OA^98^.

### Emerging approaches for DMOAD development

This section discusses new technologies and modalities emerging from the fundamental understanding of OA pathogenesis. Senescent chondrocytes accumulate in osteoarthritic cartilage and serve as a source of chronic inflammation in joints. Senolytic approaches aim to specifically remove these senescent cells. The versatility of noncoding RNAs (ncRNAs) in regulating a broad range of targets has stimulated the recent focus on RNA therapeutics, and there are now several FDA-approved RNA-based therapeutics in the pharmaceutical market. These therapeutics involve small interfering RNAs (siRNAs), microRNAs (miRNAs), or antisense oligonucleotides (ASOs) and will serve as new modalities of DMOADs, enabling the modulation of previously undruggable targets in joint tissues.

### Targeting cellular senescence

Cellular senescence refers to a state in which the cell cycle is irreversibly arrested^99,100^. In cartilage, oxidative stress associated with aging and mechanical overload mainly cause the accumulation of senescent chondrocytes^22^. Senescent chondrocytes trigger the formation of an arthritic joint microenvironment through the secretion of pro-inflammatory cytokines and proteases, which are referred to as senescence-associated secretory phenotype (SASP) factors and collectively accelerate osteoarthritic cartilage degeneration and synovial inflammation^2,22,100,101^. Two possible strategies to modulate the detrimental effects of senescence involve the use of senolytics that selectively eliminate senescent cells^100,102–108^ and senomorphics (i.e., senostatics) that abrogate the inflammatory senescent secretome^102^. UBX0101, developed as the first in-class small molecule sensitizing the senolysis of senescent chondrocytes, has shown positive results in a posttraumatic OA mouse model^100^. This senolytic drug, which decouples p53 from the MDM2-mediated degradation pathway, was tested in a phase I, double-blind, randomized, placebo-controlled trial involving 48 OA patients (NCT03513016). The clinical outcome showed a reduction in OA-associated pain without notable adverse events when UBX0101 was administered at high doses of 1.0–4.0 mg^109^. Unfortunately, the recently completed phase II trial (NCT04129944) with 180 patients did not demonstrate sufficient clinical efficacy in terms of joint pain relief.

Navitoclax (ABT-263), the most well-established senolytic drug, inhibits Bcl-2 and Bcl-xL and has been shown to attenuate OA manifestations, including cartilage degeneration and subchondral bone sclerosis, in a posttraumatic OA rat model^107^. Interestingly, neither UBX0101 nor navitoclax injection exerted any protective effect against age-associated OA in mice, whereas the combination of these two drugs ameliorated OA progression in aged animals^110^. It appears that senolytic strategies should be refined before they can be used in the clinic. Senomorphics, which modulate the phenotypes of senescent cells without killing them, may serve as alternative options to eliminate SASP factor expression and thereby abrogate the detrimental effects of senescent chondrocytes on OA development.

### RNA-based therapeutics

ncRNAs have emerged as regulators of inflammation^111,112^, chondrocyte apoptosis^113^, and ECM degradation^114,115^, which are related to OA-pathogenic mechanisms. To date, more than 50 ncRNAs, including circular RNAs, long noncoding RNAs (lncRNAs), and miRNAs, have been reported to be differentially regulated in OA, affecting the onset and progression of the disease^111,116,117^. RNA therapeutics have multiple benefits over traditional small-molecule- or antibody-based approaches, including versatility in their design to modulate target gene expression^118^. RNA therapeutics can be subcategorized into three major groups: siRNAs, miRNAs, and ASOs. These three groups use different mechanisms of action to silence their target genes but share common challenges in their clinical use: mainly in vivo delivery and stability issues^119^. Although RNAs are widely used to modulate target gene expression in vitro, their low stability and delivery efficiency in vivo limit their use as therapeutic agents^120^. The recent breakthrough in lipid nanoparticle (LNP) formulations has dramatically improved both the stability and delivery of RNA molecules, resulting in the first FDA-approved siRNA therapeutic in 2018^121^.

MMP-13 and ADAMTS-5, two critical catabolic enzymes responsible for the degradation of type II collagen and aggrecan, respectively, have been the prime targets of RNA-based therapies. Hoshi et al. examined the effect of chemically modified *Mmp13* or *Adamts5* siRNA, alone or in combination, in a posttraumatic OA mouse model^122^. Significant improvements in OA manifestations were observed in all three siRNA-treated groups (*Mmp13* siRNA alone, *Adamts5* siRNA alone, or combination) compared with the control-siRNA group. Furthermore, the combined treatment group displayed a better therapeutic outcome than the *Adamts5*–siRNA-only group.

The NF-κB pathway is the most well-known regulatory pathway governing inflammatory responses in OA^14,123,124^. Intra-articular delivery of a peptide nanoparticle containing an NF-κB siRNA alleviated cartilage degradation and synovitis in a surgically induced OA model^125^. Hypoxia-inducible factor-2α (HIF-2α) is another key transcription factor that controls the collective expression of matrix-degrading enzymes during OA development^126,127^. Intra-articular injection of an *Epas1*-targeting siRNA encapsulated in LNPs and the chondrocyte-affinity peptide DWRVIIPPRPSAC alleviated cartilage degeneration and synovial inflammation in a mouse model of OA^128^.

Compared with an siRNA, which is generally designed to exclusively knockdown a single target gene, a miRNA regulates the expression of hundreds of target genes simultaneously and has broader impacts on the transcriptome and chondrocyte physiology. There are currently no miRNAs in a clinical trials for OA treatment. Several miRNAs that can potentially delay osteoarthritic processes by modulating matrix degradation and synthesis or autophagy are listed in Table 1. In contrast, ASOs, which are short single-stranded oligodeoxynucleotides, can be used to degrade target RNAs that promote OA^129^. An ASO has been used to target miR-204, which suppresses the proteoglycan synthesis pathway and augments chronic inflammatory responses in senescent chondrocytes. This miR-204-targeting ASO effectively attenuated OA manifestations and pain development in a preclinical mouse model of OA^2^.

**Table 1:** List of miRNAs that inhibit osteoarthritis (OA) progression.

microRNA	Cell or tissue type	Mechanism	Reference
miR-132-3p	MSCs	Ectopic expression of miR-132-3p increases proteoglycan accumulation and the expression of aggrecan, type II collagen, and SOX9.	^133^
miR-107	Chondrocytes	miR-107 suppresses chondrocyte apoptosis and upregulates the expression of type II collagen while downregulating IL-1β and MMP-13.	^134^
miR-140-3p	Chondrocytes, MSCs	miR-140-3p ameliorates OA progression and promotes chondrogenesis by targeting *CXCR4*.	^135^
miR-140-5p/149	Chondrocytes	miR-140-5p/149 targets *Fut1* to promote chondrocyte proliferation and autophagy.	^136^
miR-93-5p	Chondrocytes	miR-93-5p targets *Tcf4* and the lncRNA *CASC2* and promotes chondrocyte viability by suppressing apoptosis and the expression of *Mmp3* and -*13*.	^137^
miR-335-5p	Chondrocytes	miR-335-5p alleviates the inflammatory responses in chondrocytes by upregulating autophagy-related factors (Beclin-1, ATG5, and ATG7).	^138^
miR-106a-5p	Articular cartilage	miR-106a-5a suppresses OA by targeting *Glis3*.	^139^
miR-9-5p	Chondrocytes	miR-9 promotes chondrocyte proliferation and anti-apoptotic responses by targeting the NF-κB pathway.	^140^
miR-502-5p	Chondrocytes	miR-502-5p suppresses IL-1β-induced apoptosis by targeting *TRAF2*.	^141^
miR-145	Chondrocytes	miR-145 targets *MKK4* and downregulates matrix-degrading enzymes (MMP-3, MMP-13, and ADAMTS-5).	^142^
miR-26a/26b	Chondrocytes	miR-26a/26b suppresses IL-1β-induced matrix degradation by targeting *FUT4*.	^143^
miR-411	Chondrocytes	miR-411 downregulates MMP-13, upregulates type II collagen, and induces autophagy in chondrocytes.	^144,145^
miR-27a	Synoviocytes, chondrocytes	miR-27a inhibits synovial angiogenesis and chondrocyte apoptosis by inhibiting *PLK2* and promotes autophagy.	^146,147^
miR-27b	Chondrocytes	miR-27b downregulates *MMP13*.	^114^

*MSCs* mesenchymal stem cells.

### Challenges and future directions of newly developed drugs

An important aspect of OA treatment is the consideration of diverse clinical syndromes and pathological conditions associated with stages of disease progression^130–132^. There is emerging evidence of the heterogeneity and complexity of OA pathogenesis, which urges modifications to the current “one-fits-all” treatment guidelines. Distinct molecular-level mechanisms are being rapidly elucidated to account for the diversity of OA-associated symptoms and pathogenesis. Therefore, it is urgent to establish guidelines for personalized OA treatments.

There is another vital need for the development of biomarkers that enable the early diagnosis of OA. Many of the developed DMOADs aim to delay degenerative processes in articular cartilage. Evidently, these approaches can be particularly effective in treating patients in the early stage of OA when significant cartilage remains, rather than in the late stage of OA. Therefore, when coupled with early OA diagnosis, DMOADs can fully exert their designated effects, ensuring a superior prognosis in patients with OA.

## Conclusions

With advances in the understanding of the basic molecular mechanisms underlying OA pathology, multiple DMOADs have been developed, resulting in several promising outcomes from clinical trials. In this review, we discussed multiple DMOAD options, such as those targeting inflammation, matrix-degrading enzymes and the Wnt pathway to ameliorate the degradation of cartilage matrix. Several regenerative DMOADs have shown promise in promoting the chondrogenic differentiation of stem cells and the reconstruction of cartilage matrix. Senolytic/senomorphic strategies and RNA therapeutics have been suggested to be new modalities of DMOADs, enabling the modulation of previously undruggable targets in joint tissues. DMOADs have certainly reached the point of clinical application. Their ultimate approval and availability on the pharmaceutical market are coming and will aid in the treatment of one of the most devastating joint diseases.
